# Intraoperative Findings in Total Hip Arthroplasty Leading to a Diagnosis of Alkaptonuria in a Patient With Severe Hip Osteoarthritis Initially Attributed to Rheumatoid Arthritis: A Case Report

**DOI:** 10.7759/cureus.82210

**Published:** 2025-04-13

**Authors:** Ioannis K Tzellios, Stefanos C Papageorgiou, Fotios A Tilkidis, Emilios E Pakos

**Affiliations:** 1 Department of Orthopaedic Surgery and Traumatology, University Hospital of Ioannina, Ioannina, GRC

**Keywords:** hip arthropathy, medical history, ochronosis, poor assessment / misdiagnosis, total hip arthroplasty (tha), alkaptonuria

## Abstract

Alkaptonuria is a rare inherited condition caused by elevated levels of homogentisic acid, which confers a characteristic dark color on tissues like cartilage and bones over time, a process known as ochronosis. We present the case of a 62-year-old male patient with a presumed diagnosis of rheumatoid arthritis and severe pain and stiffness in his right hip. Plain radiographs revealed severe right hip osteoarthritis. The patient underwent total hip arthroplasty (THA) without any perioperative complications. The intraoperative findings (black ring-like spot at the femoral head and also at the cartilage and acetabulum) and a more detailed medical history taken postoperatively raised suspicions of alkaptonuria, which was confirmed with the specific exam for alkaptonuria (homogentisic acid levels evaluation in a 24 h urine sample). This report underscores the importance of a thorough medical history and clinical examination of these patients to avoid inaccurate diagnosis and administration of inappropriate therapeutic schemes.

## Introduction

Alkaptonuria, also referred to as black bone disease or AKU, was the first condition shown to follow the traditional Mendelian recessive inheritance pattern. Sir Archibald Garrod initially identified it as one of the four inborn errors of metabolism in 1902 [1]. Its worldwide prevalence is estimated to range from 1:200.000 to 1:1.000.000 births. It is more prevalent in some parts of Slovakia and the Dominican Republic [2]. This disorder is caused by an autosomal recessive mutation that is located on chromosome 3 between regions 3q21 and q23, where the homogentisate 1,2-dioxygenase (HGD) gene is found [1,3]. Individuals with this disease have been found to have over 80 mutations in the HGD gene [1,3]. This enzyme, which is mostly produced in the liver and kidneys, converts homogentisic acid (HGA) into maley-lacetoacetic acid; if it is absent, HGA accumulates in different parts of the body at a rate that is more than 2000 times higher than usual [1,3].

Part of the excess HGA is excreted through the urine, which turns dark when exposed to air [3]. Homogentisic aciduria can also result from alkalization [1,3]. After first oxidation and deposition within the connective tissue, the HGA eventually transforms into ochronosis, a permanently melanin-like pigment [1,3]. Over time, this blue-black pigmentation causes early-onset osteoarthritis and black, brittle bones and cartilage, while it often manifests after the age of 30 years [1,3]. Simple benign alkaptonuria develops into alkaptonuric ochronosis, which results in the most severe symptom: ochronotic arthropathy (OA) [4]. Tissue degeneration results from pigment deposition in ligaments, elastic cartilages, chondrocytes, and the matrix of articular cartilage [5]. The thoracolumbar spine's stiffness and discomfort are usually the first clinical signs of OA [5]. The resulting articular degeneration most frequently affects the knee, hip, and shoulder joints [5].

The accumulation of HGA in hyaline cartilage, tendons, ligaments, sclera, skin, heart valves, cartilage of the nose and ears, renal tubule epithelial cells, pancreas, central nervous system, endocrine organs, respiratory organs, and arteries has been associated with other symptoms [6]. The diagnosis of alkaptonuria can be confirmed by the amount of urine HGA count and a thorough physical examination [6]. Alkaptonuria still has no recognized viable treatment [7]. The management of OA is symptomatic and similar to that of a typical arthropathy [7]. However, surgical treatment is warranted in cases of severe degenerative arthropathy [7].

In this report, we discuss an uncommon case of alkaptonuria in a 62-year-old male patient with a presumed diagnosis of rheumatoid arthritis who underwent total hip arthroplasty (THA); the correct diagnosis was suspected based on the intraoperative findings and confirmed after a thorough postoperative medical history and the proper laboratory exam. This case has been reported in line with the Surgical Case Report (SCARE) criteria.

## Case presentation

**Figure 1:**
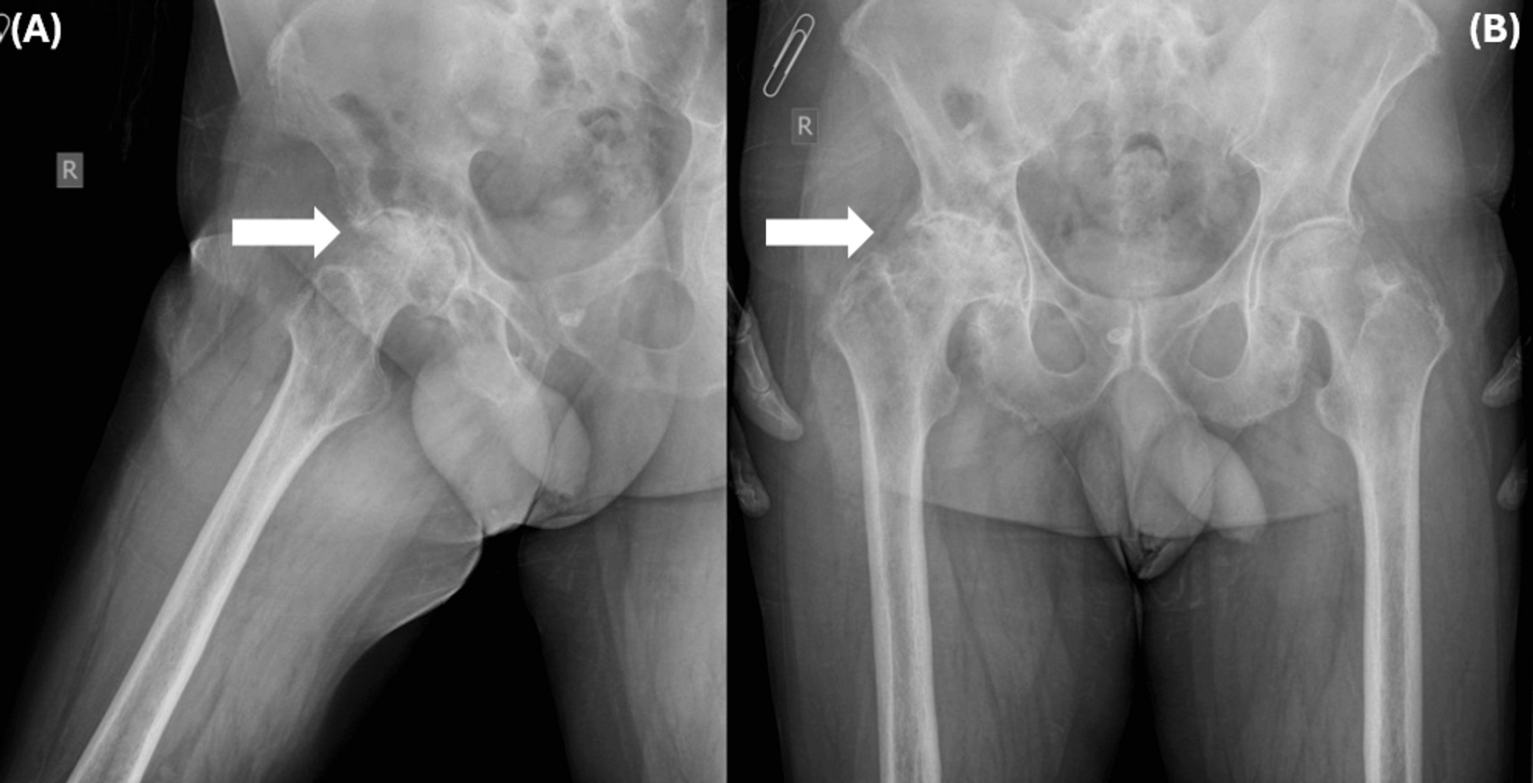
Preoperative radiological examination (A) Oblique view of the right hip and (B) anteroposterior view of the pelvis indicating severe hip osteoarthritis (white arrows)

A 62-year-old Caucasian male presented at the Orthopaedic Outpatient Department due to severe hip pain and discomfort during simple daily tasks for the last two years. His medical history included hypertension, dyslipidemia, and rheumatoid arthritis, which had been diagnosed nine months before by a rheumatologist and since then treated with methotrexate. Physical examination showed a limited range of motion of the hip and tenderness. Plain radiographs of the pelvis in anteroposterior (AP) view and the right hip in oblique view revealed severe osteoarthritis of the right hip (Figure 1). Surgical treatment (THA) was proposed as the best treatment option.

**Figure 2:**
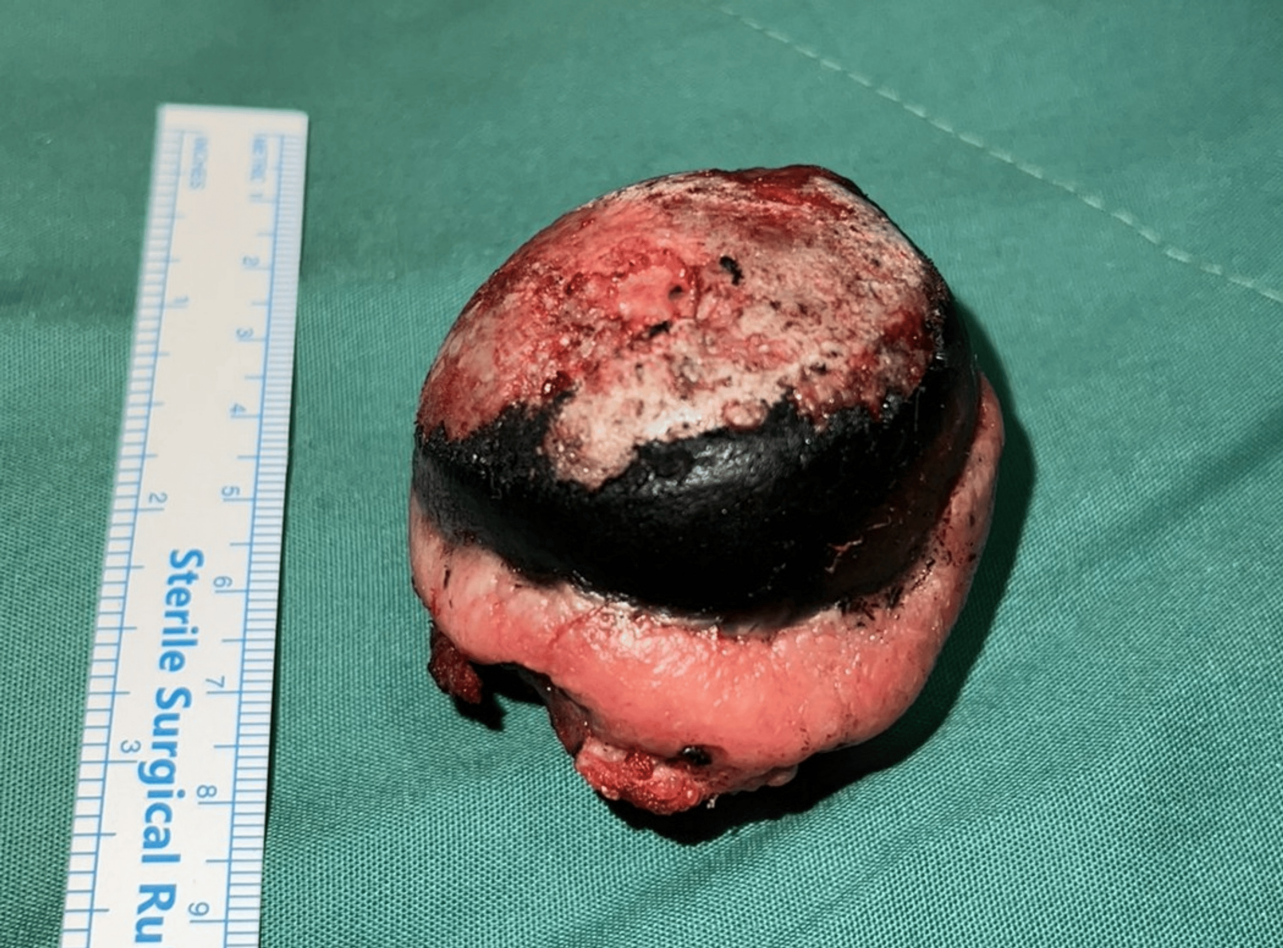
Black ring-like spots on the resected femoral head, typical in ochronotic arthritis

**Figure 3:**
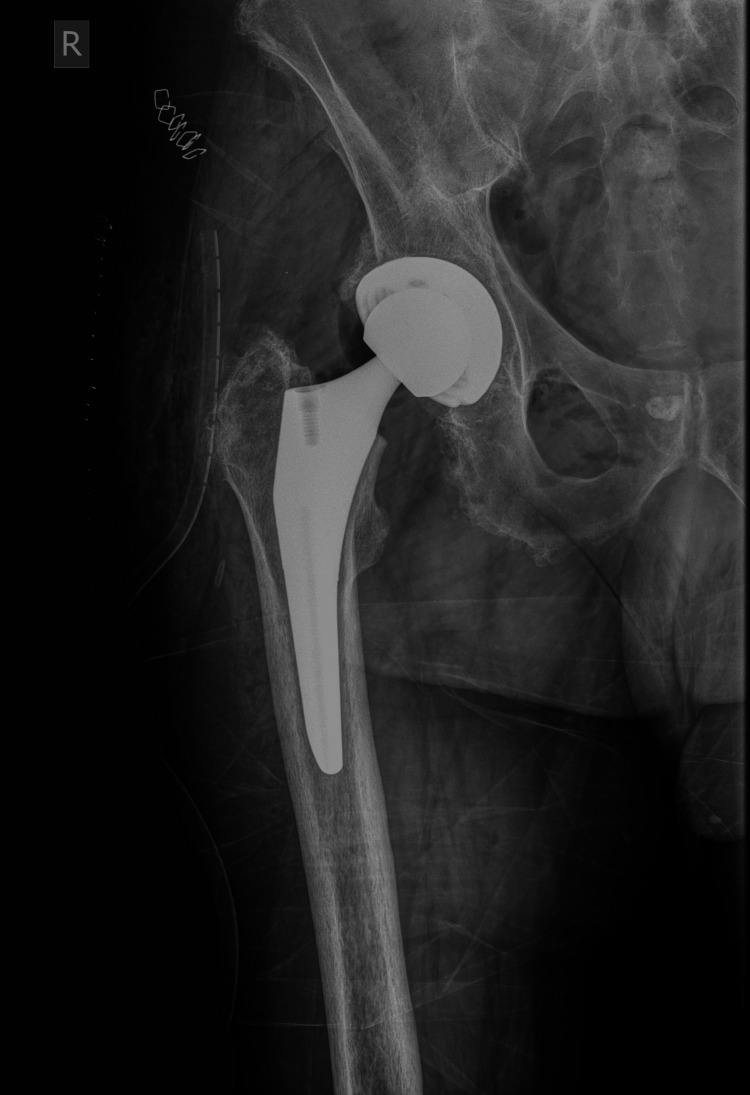
Postoperative radiograph of the right hip in AP view AP: anteroposterior

Surgery was performed under spinal anesthesia with the patient positioned in the left lateral decubitus position. The hip joint was accessed with a typical posterolateral approach. After the hip dislocation, unusual black ring-like spots were noticed at the femoral head and the acetabulum (Figure 2). These findings raised concerns for some underlying disorder, particularly alkaptonuria. The initial plan was maintained, and THA with press-fit fixation of its components was completed (Figure 3). Tissue samples from the femoral head and the capsule were sent for histopathologic analysis.

**Figure 4:**
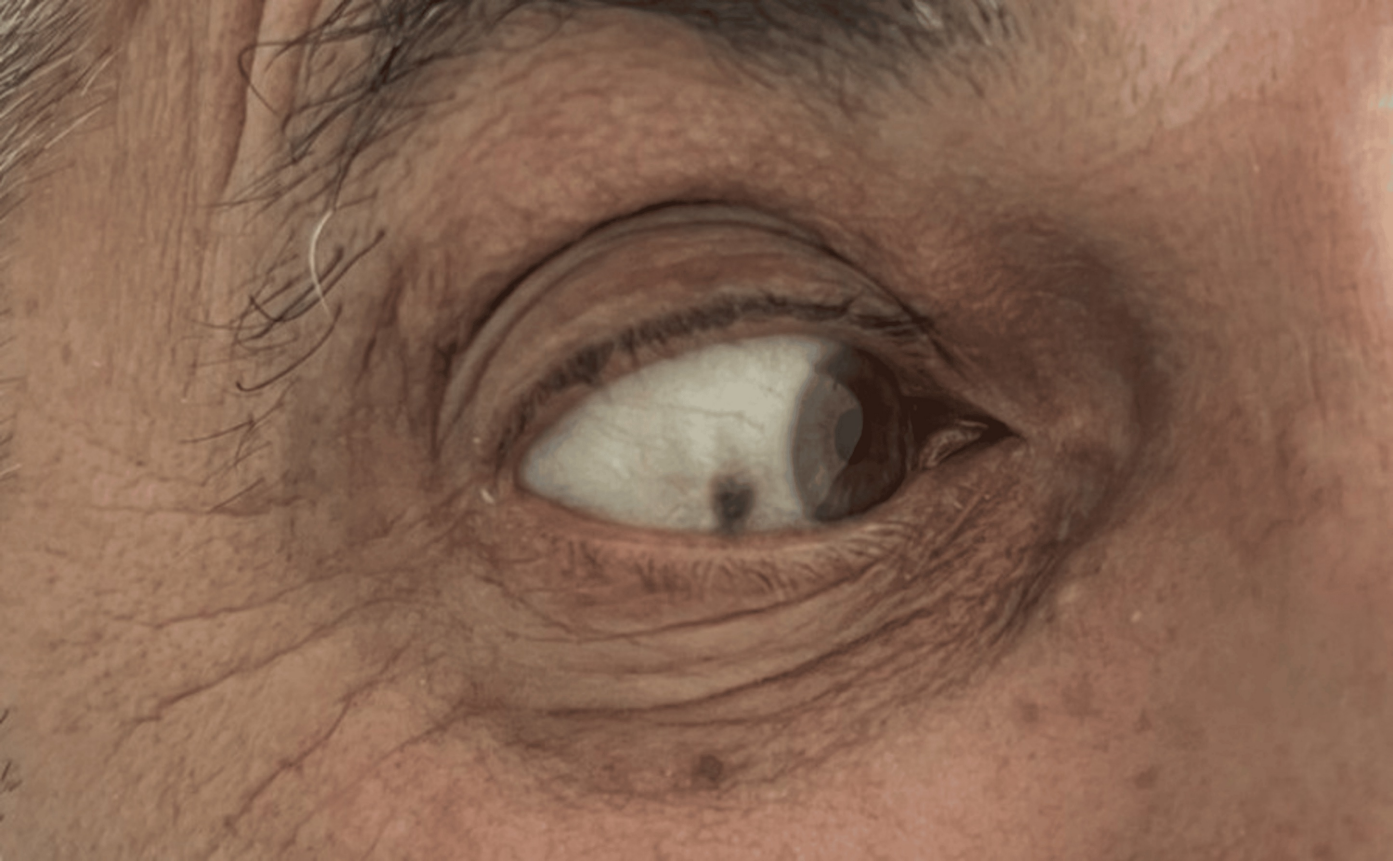
Characteristic sclera pigmentation of patient’s right eye

After the surgery, the patient confirmed that he had never been diagnosed with alkaptonuria in the past. Furthermore, he was examined for other signs while his medical history was revisited. He had black patches in his sclerae in both eyes (Figure 4) and reported blackish discolorations of his urine, two symptoms consistent with the diagnosis of alkaptonuria. He also reported that a few months ago, during the exhumation of his mother’s remains, the personnel of the graveyard had been surprised by the black color of her bone remains. A 24 h urine sample was sent to evaluate the levels of HGA, which is the main diagnostic method for alkaptonuria. After two days, the results of the exam revealed high levels of HGA (1.4 gr/24 h; normal value: <0.1 gr/24 h), consistent with the initial intraoperative suspicion. Urinary HGA was measured by gas chromatography. Histopathologic analysis revealed deposition of black-coloured granules over the pigmented areas of the femoral head and an inflammed thickened capsule.

## Discussion

Patients with alkaptonuria are asymptomatic in 40% of cases. Clinical symptoms typically appear in the third or fourth decade of life [1,7,8]. Common musculoskeletal symptoms include kyphoscoliosis, spondylosis, fractures, osteopenia, ruptured tendons, and secondary osteoarthritis of big weight-bearing joints [1,6]. Calcification of the intervertebral disc, menisci, pubic symphysis, nasal cartilages, pinnae, heart valves, and major vessels are all distinctive radiographic characteristics [1,6]. After their third decade of life, one-third of patients experience secondary osteoarthritis of the major joints, such as the hip, knee, and shoulder [1]. Males are more frequently affected than females [1]. This arthropathy results from alterations in the biochemical nature of articular cartilage, which causes it to become fragile [3]. Similar to primary osteoarthritis, there is a significant narrowing of the joint space [3]. Compared to radiological symptoms, the clinical aspects of pain and limping are more evident [3].

Ochronotic arthropathy is typically managed with conservative treatment [1,7,8]. The condition is often identified intraoperatively during joint replacement surgery based on dark pigmentations of the cartilage and synovium [1,2]. In our patient as well, ochronosis was suspected during arthroplasty of the right hip due to the dark coloration of the femoral head and joint capsule, which was subsequently verified by the histological examination. There is scarce research on total hip replacement for ochronotic arthropathy. The survival rate of prostheses is equivalent to that of arthroplasty performed for other causes, according to Spencer et al.'s analysis of 11 different arthroplasties performed on three ochronotic patients over a 12-year follow-up period [9]. After performing an uncemented hip replacement and a cemented knee replacement on a patient with ochronosis, Harun et al. concluded that arthroplasty provides great results for individuals with ochronosis-related degenerative arthritis [7]. Makela et al. treated a severe case of ochronotic arthropathy with bilateral hip replacements within eight months, and the patient's hips were free of any discomfort and showed improved mobility at follow-up [10].

Fernando et al. suggested removing the capsule entirely following a hip replacement to stop the disorder process from recurring [11]. Results of arthroplasty in 12 ochronotic hips with a 3-24-year follow-up were published by Pachore et al. [12]. They advised against a total synovectomy after noticing higher blood loss in these patients [12]. Couto et al. advocate capsular removal as a contracted pathological capsule will limit hip mobility [13]. In our case, we resected the capsule entirely without any significant blood loss. Our patient did not present any hip mobility restrictions during the follow-up. Because of the potential inflammatory nature of arthritis, Lee et al. recommended a cemented prosthesis on total knee arthroplasty in ochronotic patients [14].

Τhere is little data to compare the outcomes of cementless versus cemented arthroplasty of the hip. In line with our findings, Couto et al., in their report of hip arthroplasty with perioperatively diagnosed alkaptonuria, proceeded with a cementless component as the bone seemed to be in good condition macroscopically [13]. During reaming, Cebesoy et al. noticed that the hip joint's acetabular and femoral bone stock was of poor quality [15]. Considering the acetabular sclerosis during reaming, Pachore et al. advised the use of screws alongside the cup in their case report [12].

## Conclusions

Alkaptonuria can cause joint pain and stiffness, which may be easily confused with conditions like rheumatoid arthritis or osteoarthritis. Our patient’s hip joint degeneration was caused by alkaptonuria. The scleral pigmentation and the blackish urine samples were not taken into account in the first evaluation; however, after the intraoperative findings, the pieces of the puzzle started falling in place, while the specific urine test led to the diagnosis of alkaptonuria. This case illustrates that ignoring certain small clinical findings may lead to a misdiagnosis in these patients, causing them to undergo an incorrect treatment pathway.
